# Intermediate metabolites of the pyrimidine metabolism pathway extend the lifespan of *C. elegans* through regulating reproductive signals

**DOI:** 10.18632/aging.102033

**Published:** 2019-06-21

**Authors:** Qin-Li Wan, Xiao Meng, Xiaodie Fu, Bohui Chen, Jing Yang, Hengwen Yang, Qinghua Zhou

**Affiliations:** 1 The Center for Precision Medicine of First Affiliated Hospital, Biomedical Translational Research Institute, Jinan University, Guangzhou 510632, China

**Keywords:** *Caenorhabditis elegans*, longevity, pyrimidine metabolism, reproductive signals

## Abstract

The pyrimidine metabolism pathway has important biological functions; it not only maintains appropriate pyrimidine pools but also produces bioactive intermediate metabolites. In a previous study, we identified that the pyrimidine metabolism pathway is associated with aging regulation. However, the molecular mechanism by which the pyrimidine metabolism pathway regulates aging remains unclear. Here, we investigated the longevity effect of pyrimidine intermediates on *Caenorhabditis elegans* (*C. elegans*). Our results demonstrated that the supplementation of some pyrimidine intermediates could extend the lifespan of *C. elegans*. In addition, the RNAi knockdown of essential enzymes involved in pyrimidine metabolism could also significantly affect lifespan. We further investigated the molecular mechanism by which a representative intermediate metabolite, thymine, extends the lifespan of worms and found that thymine-induced longevity required the nuclear receptors DAF-12 and NHR-49, and the transcription factor DAF-16/FOXO. Further pathway analysis revealed that the longevity effect of thymine depended on the inhibition of reproductive signals. Additionally, we found that other pyrimidine intermediates functioned in a manner similar to thymine to prolong lifespan in *C. elegans*. Taken together, our results revealed that pyrimidine intermediates increased lifespan by inhibiting reproductive signals and subsequently inducing the function of DAF-12, NHR-49 and DAF-16 in *C. elegans.*

## INTRODUCTION

Aging is universally characterized by a progressive loss of physiological integrity, leading to organ failure [1, 2]. This deterioration is the principal risk factor for numerous chronic diseases, such as type 2 diabetes, cancer, cardiovascular disorders, and neurodegenerative diseases [3]. Therefore, interventions to prevent or attenuate age-related degeneration are an unmet need. To date, some drugs that can affect lifespan and healthspan have been found, such as rapamycin and metformin, which increase lifespan in multiple species [4, 5]. Both drugs have been proven to be relatively safe over decades of clinical use; however, side effects are sometimes observed. Therefore, the discovery of new anti-aging drugs is urgent. Endogenous metabolites that can regulate lifespan have considerable advantages because they have limited toxicity and side effects. Recently, several metabolites have been identified to modulate aging. For example, one study found that dietary supplementation with the nematode eicosapentaenoyl ethanolamide resulted in a significant reduction in lifespan and stress tolerance [6]. Another study reported that oxaloacetate supplementation increased lifespan in a DAF-16-dependent manner in *C. elegans* [7]. Additionally, a previous study also reported that dietary supplementation with the metabolite α-ketoglutarate lengthened lifespan by inhibiting ATP synthase and TOR signaling [8].

Intermediates of pyrimidine metabolism are essential biomolecules, as they participate in diverse cellular functions, such as the synthesis of DNA, RNA, lipids, and carbohydrates [9]. Previous studies have reported that pyrimidine metabolism disruption causes some diseases, such as Alzheimer's disease, immunodeficiency, and growth retardation [10, 11], and the downregulation of pyrimidine metabolism was linked to aging in mice [12]. In our previous study, we reported that the levels of pyrimidine intermediates were significantly decreased in aged worms, suggesting that pyrimidine metabolism is related to the aging of *C. elegans* [13]*.* However, the mechanism by which the pyrimidine metabolism pathway regulates longevity remains unknown.

In this study, we used *C. elegans* as a model organism to explore the mechanism by which pyrimidine metabolism regulates aging. *C. elegans* is an excellent model for studying the molecular mechanism of longevity modulation. Genetic studies in *C. elegans* led to the identification of numerous molecular pathways that regulate longevity, including the insulin/insulin-like growth factor (IGF), target of rapamycin (TOR), germline signaling pathways, proteostasis, and mitochondrial function pathways [14–17]. These genetic pathways identified to regulate longevity are evolutionarily conserved, from yeast to human. Here, we characterized the biological effects of intermediates of the pyrimidine pathway, including thymine, orotate, β-aminoisobutyrate, uridine and cytidine. We found that pyrimidine intermediates could significantly prolong the lifespan of *C. elegans* through the inhibition of reproductive signaling, the activation of the nuclear receptors NHR-49 and DAF-12, and the upregulation of the transcription factor DAF-16.

## RESULTS

### Intermediate metabolites in the pyrimidine metabolism pathway increase the lifespan of *C. elegans*

**Figure 1:**
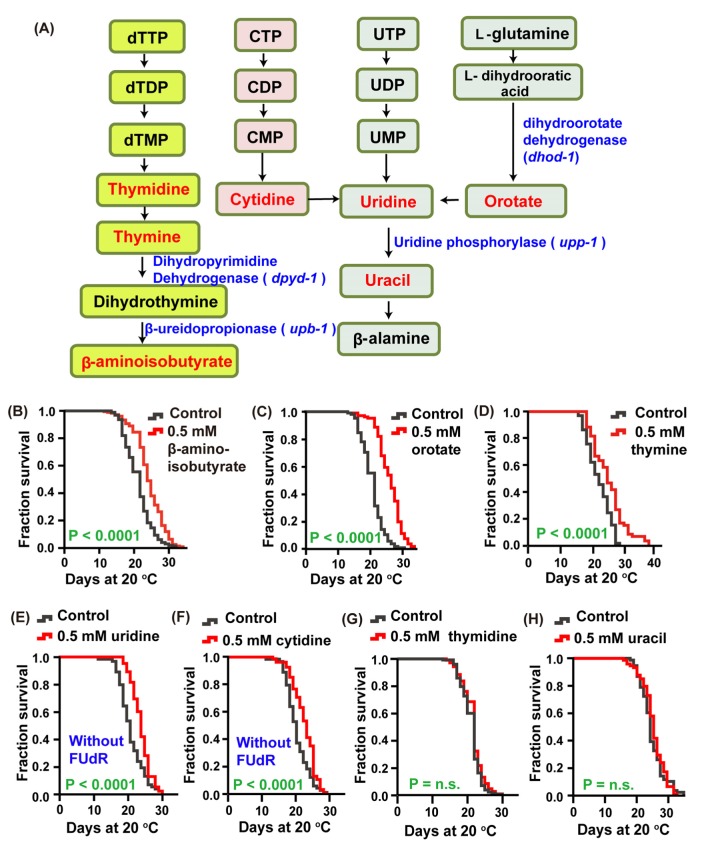
**The intermediate metabolites of the pyrimidine metabolism pathway extended the lifespan of *C. elegans*.** (**A**) Schematic representation of the pyrimidine metabolism pathway. (**B**–**H**) Lifespan of worms treated with 0.5 mM (**B**) β-aminoisobutyrate (red), (**C**) orotate (red), (**D**) thymine (red), (**E**) uridine (red), (**F**) cytidine (red), (**G**) thymidine (red), (**H**) uracil (red), and vehicle (gray) at 20 °C. The treatments were administered beginning on day 1 of adulthood. Lifespan was analyzed using the Kaplan-Meier test, and *P* values were calculated using the log-rank test; no significant difference was abbreviated as n.s. Data are representative of at least two independent experiments, and lifespan values are listed in Supplementary Table 1.

To dissect the mechanism by which the pyrimidine metabolism pathway (Figure 1A)regulates lifespan, we detected the effect of intermediate metabolites in this metabolic pathway on the lifespan of *C. elegans* by feeding worms these endogenous small molecules. We found that feeding animalsthymine, β-aminoisobutyrate, orotate, uridine and cytidine at a concentration of 0.5 mM on *Escherichia. coli* OP50, which were heat-inactivated to avoid the catalysis of compounds by bacteria, could significantly extend lifespan by 15.2, 9.69, 14.8, 9.83, 7.87%, respectively, compared with the nontreated control (Figure 1B–1F). By contrast, treatment with uracil and thymidine at the same concentration had no effect on lifespan (Figure 1G–1H). These data suggest that the pyrimidine metabolism pathway plays an important role in regulating the lifespan of *C. elegans.* We also found that worms treated with 0.5 mM uridine and cytidine exhibited 5-fluro-2′-deoxyuridine (FUdR) resistance, as 10 μM FUdR could not prevent self-fertilization. Therefore, in all subsequent lifespan analyses using uridine and cytidine, animals were maintained on the plates without FUdR.

### Essential genes in the pyrimidine metabolic pathway regulate the aging of *C. elegans*

**Figure 2:**
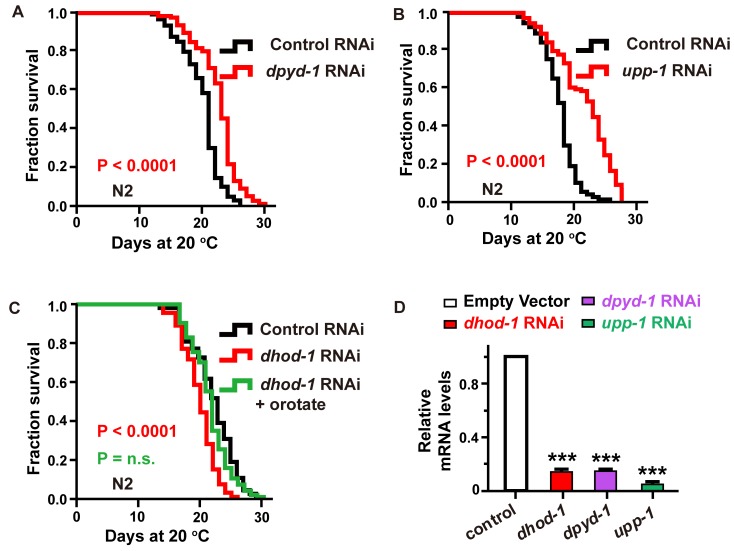
**Characterization of the genes involved in the pyrimidine metabolism pathway as aging-related genes.** (**A**–**C**) The effect of (**A**) *dpyd-1* RNAi (red) and (**B**) *upp-1* RNAi (red) on lifespan. (**C**) Lifespan of wild-type N2 exposed to control, *dhod-1* RNAi, and *dhod-1* RNAi with orotate supplementation. Lifespan values of repetitions are listed in Supplementary Table 1. (**D**) *dhod-1*, *dpyd-1*, and *upp-1* RNA levels in whole worms after treatment of *C. elegans* with RNAi against *dhod-1*, *dpyd-1* and *upp-1*, respectively, versus control RNAi. (mean ± SD, n=3, *** *P*<0.001, Student’s t test).

In *C. elegans*, *dpyd-1* encodes the *C. elegans* ortholog of human dihydropyrimidine dehydrogenase (DPYD), which is the first and rate-limiting enzyme for the metabolism of thymine to 5,6-dihydrothymine [18]. We detected that knockdown of the *dpyd-1* gene by RNAi, which dramatically decreased the mRNA level, extended the lifespan of worms (Figure 2A and 2D).

Similarly, the *C. elegans* UP homologous protein (UPP-1) exhibits both uridine and thymidine phosphorylase activity [19]. We found that lifespan was significantly extended (Figure 2B and 2D) following the exposure of *C. elegans* to *upp-1* RNAi bacteria.

Furthermore, the *C. elegans* DHODH (dihydroorotate dehydrogenase (quinone)) homologous protein DHOD-1 catalyzed the transition of dihydroorotate to orotate [20]. We observed that the lifespan of *C. elegans* was significantly decreased following exposure to *dhod-1* RNAi (Figure 2C and 2D). Additionally, we found that the decreased lifespan caused by *dhod-1* impairment could be rescued by supplementation with 2 mM orotate (Figure 2C). In previous experiments, metabolites were applied at a concentration of 0.5 mM when feeding heat-inactivated OP50. However, for RNAi experiments, in which bacteria cannot be inactivated, 2 mM was used to compensate for the loss caused by the portion of treatments metabolized by live HT115 bacteria.

However, the RNAi knockdown of *upb-1*, a gene that catalyzes the transition of dihydrothymine to β-aminoisobutyrate [21], had no effect on the lifespan of treated worms (Supplementary Figure 1A and 1B, Supporting information). One possibility is that *upb-1* is involved in several metabolic pathways, and its loss of function may cause some metabolite variations instead of exclusive changes in the levels of β-aminoisobutyrate. Accordingly, these results were consistent with a direct effect of metabolites, including thymine, uridine, cytidine, and orotate, on longevity.

### Thymine regulates aging via a mechanism dependent on the transcription factor FOXO/DAF-16 and the nuclear receptors DAF-12 and NHR-49

**Figure 3:**
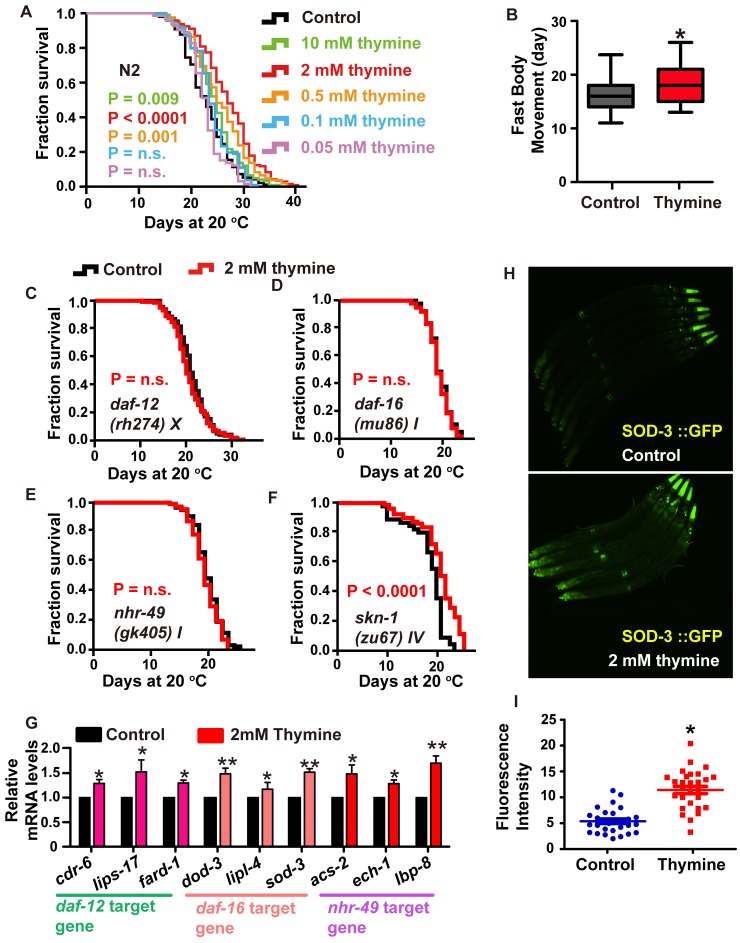
**Lifespan extension induced by thymine treatment is mediated by the nuclear receptors DAF-12 and NHR-49, and the transcription factor DAF-16/FOXO.** (**A**) Effects of thymine supplementation at increasing concentrations (0.05-10 mM) (*P* value determined by log-rank tests). (**B**) Age-related movements of worms treated with 2 mM thymine and vehicles. Data are the mean ± SD; * *P* <0.05 (Student’s t test). (**C**–**F**) Lifespan analysis in (**C**) *daf-12 (rh274)*, (**D**) *daf-16 (mu86)*, (**E**) *nhr-49 (gk405)*, and (**F**) *skn-1 (zu67)* worms treated with 2 mM thymine (red) and vehicle (black), on heat-inactivated *E. coli* OP50 (*P* value by log-rank test). Lifespan values of replicated experiments are summarized in Supplementary Table 1. (**G**) QPCR analysis of the mRNA level of target genes of *daf-12*, *daf-16* and *nhr-49* in *C. elegans* treated with 2 mM thymine versus control. Data are the mean ± SD, n=3, * *p*<0.05 (Student’s t test). (**H**–**I**) Images and quantification of GFP fluorescence. *p < 0.05; n=30 (Student’s t test).

Thymine is a representative intermediate metabolite in the pyrimidine metabolism pathway, and it is a stable molecule that had the best effect on lifespan extension among all the metabolites that benefited the longevity of *C. elegans.* Therefore, we chose thymine to systematically explore the molecular mechanism of the pyrimidine pathway. We exposed wild-type N2 worms to different concentrations of thymine and found that animals raised on plates containing 2 mM of thymine showed the largest lifespan extension of 18.3% (Figure 3A). Worms exposed to 10 mM and 0.5 mM also had moderately extended the lifespan (Figure 3A). At the lower concentrations of 0.1 and 0.05 mM, no lifespan extension was observed (Figure 3A). Therefore, thymine was applied at a concentration of 2 mM in all subsequent experiments.

To assess whether healthspan was affected, we measured an age-related phenotype, body movement, which is one of the most obvious behavioral abnormalities associated with nematode aging [22]. We analyzed the body movement of animals treated with or without 2 mM thymine. Our results showed that body movement progressively declined during aging, while the decrease in body movement with aging was delayed by thymine (Figure 3B).

To explore whether and to what extent known transcriptional modulators of lifespan might contribute to the longevity phenotype caused by thymine, we performed survival analyses of different mutants. SKN-1, a homolog of mammalian Nrf2 (nuclear factor-erythroid related factor 2) [23], appeared dispensable for thymine-mediated lifespan extension, as thymine could further increase the lifespan of the *skn-1* mutant; moreover, the expression of a target gene of *skn-1* (*gst-4*) and the P_gst-4_::GFP reporter were not significantly different between animals treated with or without 2 mM thymine. (Figure 3F and Supplementary Figure 2). The *C. elegans* DAF-16 [24], nuclear receptor PPARα homolog NHR-49 [25] and steroid nuclear receptor DAF-12 [26] appeared to be involved since the impairment of *nhr-49*, *daf-12* and *daf-16* abolished the lifespan extension induced by thymine (Figure 3C–3E). In addition, several major target genes of *daf-16* (*sod-3*, *dod-3* and *lipl-4*) [23, 27], *daf-12* (*card-6*, *lips-17* and *fard-1*) [28] and *nhr-49* (*acs-2*, *ech-1* and *lbp-8*) [29] were significantly increased when worms were exposed to 2 mM thymine (Figure 3G). Additionally, the expression of the P_sod-3_::GFP reporter in transgenic worms that were exposed to 2 mM thymine was significantly higher than that in nonexposed worms (Figure 3H and 3I). Taken together, these results indicated that thymine-induced lifespan extension requires the function of DAF-12, NHR-49, and DAF-16.

### The effect of thymine on lifespan extension may be conferred by a reduction in germline signaling

**Figure 4:**
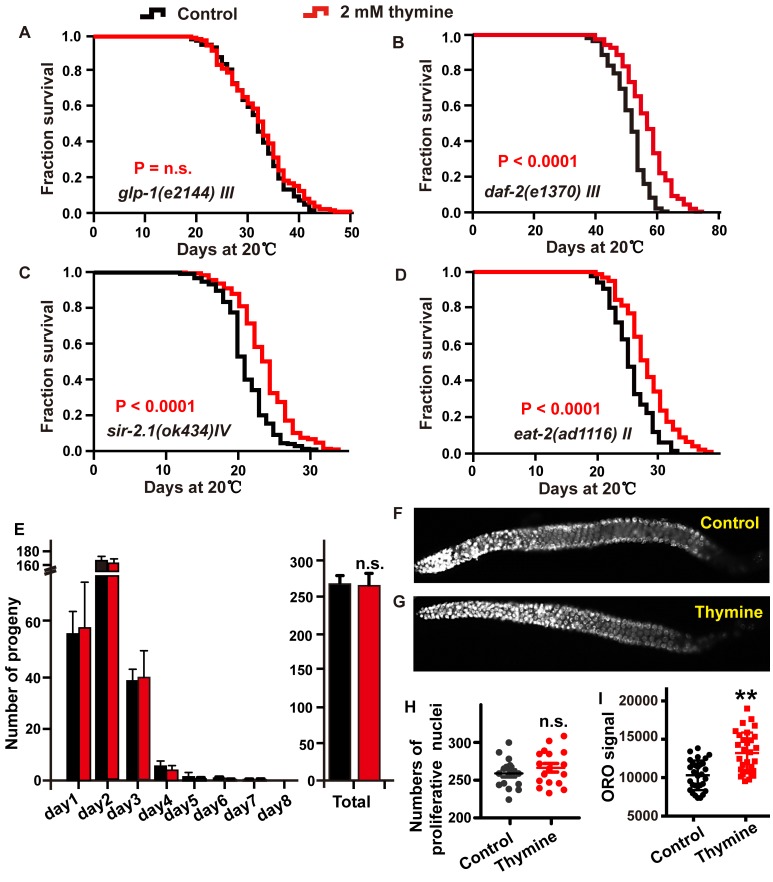
**The lifespan extension induced by thymine depends on reproductive signals.** (**A**–**D**) Lifespan analysis of (**A**) *glp-1 (e2144)*, (**B**) *daf-2 (e1370)*, (**C**) *sir-2.1 (ok434),* and (**D**) *eat-2 (ad1116)* animals treated with 2 mM thymine (red) and vehicle (black), on heat-inactivated *E. coli* OP50 (log-rank test). Lifespan values of replicated experiments are summarized in Supplementary Table 1. (**E**) The number of daily progeny and the total number of progeny of wild-type N2 worms treated with 2 mM thymine or vehicle. (**F**, **G**) DAPI-stained image of worm gonads. (**H**) Quantification of germline stem cells in wild-type N2 animals treated with 2 mM thymine or vehicle; n.s. not significant (Student’s t test). (**I**) Quantification of ORO staining. Mean ± SD.; n ≥ 30 per condition; *P* value was calculated using Student’s test; no significance is abbreviated as n.s. *p < 0.05, **p < 0.01, ***p < 0.001.

We next dissected which longevity pathway is required for the lifespan extension induced by thymine through testing its effects in corresponding mutants. The ability of the Notch signaling pathway to sense nucleotide abundance is reminiscent of *glp-1* [30], which encodes a Notch family receptor and is essential for the mitotic proliferation of germline cells [31]. The *glp-1* loss-of function (lf) mutants have obviously prolonged lifespan when maintained at the nonpermissive temperature due to failed germline proliferation, which is dependent on the transcriptional activities of DAF-16, DAF-12, and NHR-49 [16, 32, 33]. Therefore, we first tested whether the longevity benefit of thymine was mediated by the Notch signaling pathway. Treatment with 2 mM thymine failed to further increase the lifespan of *glp-1* mutants (Figure 4A), suggesting that thymine might regulate the germline signal to extend lifespan. The downregulation of germline signaling extends lifespan, usually accompanied by a decrease in the production of progeny [34]. However, we did not find that thymine decreased the daily progeny production, total progeny and germ cells of each worm (Figure 4E–4H). One possible explanation is that thymine does inhibit reproductive signals, but this inhibition does not reach a significant extent to affect the amount of eggs laid. Reduced reproductive signals is usually accompanied by a change in lipid metabolism [35]. To determine whether thymine affects fat metabolism, we used Oil-Red-O (ORO) staining. We found that wild-type animals treated with 2 mM thymine had significantly increased ORO staining (Figure 4I).

DAF-16 is downstream of several signaling pathways in addition to the reproduction signaling pathway, such as the IGF signaling pathway [24]. To explore whether thymine contributes to lifespan extension by influencing the IIS signaling pathway in a DAF-16-dependent manner, we detected the effect of thymine on long-lived mutants of the insulin-like receptor *daf-2*. We observed that the lifespan of *daf-2* was significantly increased by thymine treatment, suggesting that the lifespan extension induced by thymine might not depend on the IIS pathway (Figure 4B).

The activation of DAF-16 by thymine was reminiscent of the silent information regulator 2 (SIR2), which encodes a nicotinamide adenine dinucleotide (NAD)-dependent deacetylase. SIR-2.1 could bind to DAF-16 and extend lifespan in a 14-3-3 protein-dependent manner [36]. We investigated whether SIR-2.1 mediated the lifespan extension caused by thymine using a null mutant *sir-2.1* strain. Thymine extended the lifespan of the *sir-2.1* mutant, indicating that SIR-2.1 was dispensable for thymine-mediated lifespan extension (Figure 4C).

Dietary restriction (DR) plays a significant role in the aging process in a wide range of species. The nuclear receptor NHR-49 and the transcription factor DAF-16 [37, 38], which function as mediators of the lifespan extension induced by thymine, were activated to respond to low energy and modulate lifespan under starvation conditions. Therefore, to determine whether the ability of thymine to extend lifespan was related to an effect similar to DR, we used a pharyngeal pumping defective *eat-2* mutant, which was considered DR-constitutive due to its reduced food intake [39]. As shown in Figure 4D, thymine treatment significantly increased the mean lifespan of the *eat-2* mutant, indicating that thymine acted through mechanisms different from the DR related mechanisms to extend the lifespan of *C. elegans*.

### Other pyrimidine intermediates extend the lifespan of *C. elegans* in a similar mechanism mediated by thymine

**Figure 5:**
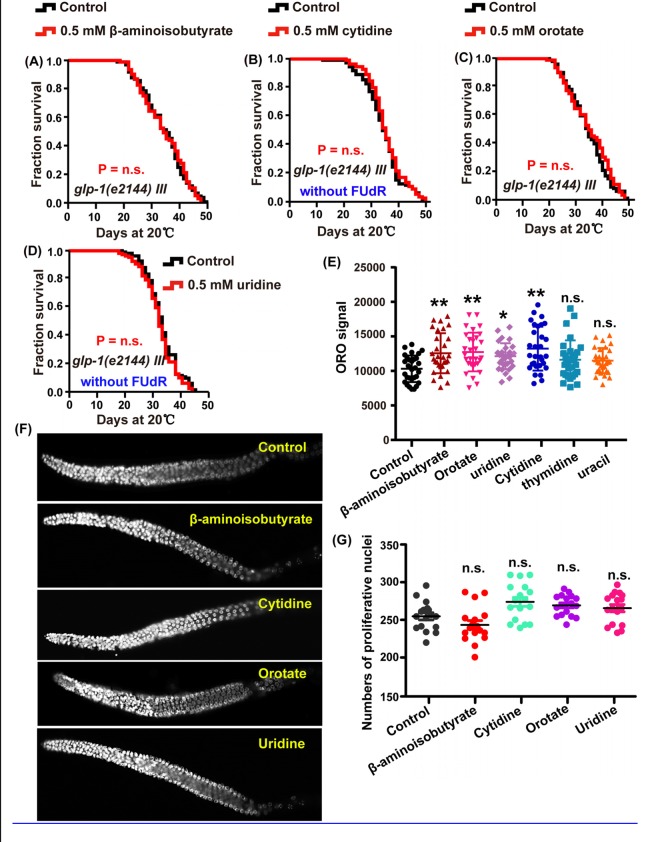
**Intermediate metabolites in pyrimidine metabolism influence the lifespan of *C. elegans* through regulating reproductive signals.** (**A**–**D**) Lifespan analysis of *glp-1 (e2144)* animals treated with 0.5 mM (**A**) β-aminoisobutyrate (red), (**B**) cytidine (red), (**C**) orotate (red), (**D**) uridine (red), and water (black) on heat-inactivated *E. coli* OP50 (*P* value by log-rank test). The replicated data are summarized in Supplementary Table 1. (**E**) Quantification of ORO staining. Mean ± SD.; n ≥ 30 (Student’s t test). (**F**) DAPI-stained image of the gonads of N2 worms treated with 0.5 mM β-aminoisobutyrate, cytidine, orotate, and uridine and the untreated control. (**G**) Quantification of the germline stem cells in wild-type N2 worms treated with or without 0.5 mM of the metabolites. *P* values were calculated using Student’s t test.

**Figure 6:**
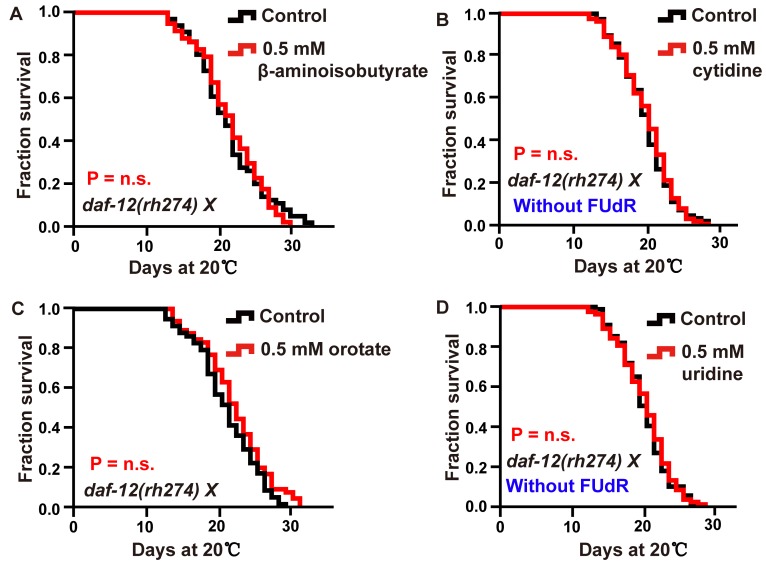
**Intermediate metabolites in pyrimidine metabolism extended adult lifespan in a DAF-12-dependent manner.** (**A**–**D**) Lifespan of *daf-12 (rh274)* animals treated with 0.5 mM (**A**) β-aminoisobutyrate, (**B**) cytidine (red), (**C**) orotate (red), (**D**) uridine (red), and vehicle (black). The *P* value was calculated by the log-rank test. Replicates of these experiments and statistical details are summarized in Supplementary Table 1.

In addition to thymine, other metabolites of the pyrimidine metabolism pathway, including β-aminoisobutyrate, uridine, cytidine and orotate could promote the lifespan of *C. elegans*. We tested whether the mechanism contributing to thymine-induced lifespan regulation was also involved in the lifespan extension induced by other pyrimidine intermediates. Our results revealed that β-aminoisobutyrate, uridine, cytidine, and orotate failed to further increase the lifespan of *glp-1* (Figure 5A–5D) and *daf-12* (Figure 6) mutants, which represent the reproduction signaling pathway. Similarly, the lipid content of wild-type worms was significantly increased when animals were exposed to 0.5 mM β-aminoisobutyrate, uridine, cytidine, and orotate (Figure 5E). Moreover, similar to the effect of thymine on reproduction, these metabolites could not affect germline stem cells of *C. elegans* (Figure 5F and 5G). Collectively, these results demonstrated that pyrimidine intermediates increased the lifespan of *C. elegans* by influencing the reproduction signaling pathway rather than by directly influencing fecundity.

## DISCUSSION

Pyrimidine metabolism has been broadly studied in many organisms. The importance of this metabolic pathway is due to the fact that they provide pyrimidine nucleosides, which are essential components of many biomolecules. Additionally, pyrimidine metabolism disorders can cause some diseases, such as orotic aciduria, which results from uridine monophosphate synthase (UMPS) deficiency [40]. Previously, a large number of studies focused on the relationship between pyrimidine and cancer. Pyrimidine derivatives have been used as anticancer agents, such as 5-fluorouracil (5-FU), which is a predrug molecule and is converted to an active drug via the pyrimidine biosynthesis pathway. Therefore, the function of this drug is dependent on the activities of the pyrimidine synthesis enzymes [41]. Moreover, our previous study suggested that pyrimidine metabolism is closely associated with aging of *C. elegans* [13]. In the present study, we screened some intermediates involved in pyrimidine metabolism and found that thymine, uridine, cytidine, orotate, and β-aminoisobutyrate could significantly increase the lifespan of *C. elegans* (Figure 1B–1F). In addition, we demonstrated an uncharacterized mechanism by which pyrimidine nucleotides could extend the lifespan of *C. elegans* by inhibiting the Notch-associated reproduction signaling pathway. These result is consistent with the analysis of concentration of pyrimidine intermediates between *glp-1* mutants and wild-type in our previous study. In that study, we found that there are many difference in concentration of pyrimidine intermediates between *glp-1* mutant and wild-type N2, such as β-aminoisobutyrate, uracil, UMP and CDP and so on [13]. However, due to the limitation of detection sensitivity, we did not detect changes in concentration of thymine, thymidine and uridine in previous study. Here, we further performed the analysis of levels of pyrimidine intermediates between *glp-1* mutant and wild-type N2, and found that the level of thymine and thymidine significantly increased in *glp-1* mutant compared with wild-type N2, those of uridine moderately increased but no significant difference (Supplementary Figure 4). In the in-depth pathway analysis, we revealed that these metabolites extend lifespan by downregulating reproductive signals and then evoking the nuclear receptors DAF-12 and NHR-49, and the transcription factor DAF-16.

Nucleotide homeostasis is tightly associated with human health. In humans, abnormally elevated concentrations of pyrimidine intermediate metabolites can lead to a high incidence of many diseases. For example, a high nucleotide supply could lead to the uncontrolled proliferation of cancer cells [42]. Increased uracil levels will result in urea cycle disorders [43]. The depletion of 5-phosphoribosyl-1-pyrophosphate (PRPP) causes orotate accumulation, which can result in secondary orotic aciduria [44]. However, pyrimidine nucleotide starvation has also been linked to some diseases, such as developmental delay, seizures, alopecia and recurrent infections [45]. In our study, we found that the elevated concentrations of pyrimidine metabolism intermediates induced by exogenous supplementation could significantly increase lifespan. The mechanism underlying this complicated regulatory feedback loop in worms that modulates internal nucleotide homeostasis remains to be explained. Based on a previous study [30], worms sense nucleotide levels through the Notch signaling pathway. When the level of nucleotides was low, animals shut down germline proliferation to protect both the mother and the progeny from irreversible and deleterious effects resulting from the exhaustion of the limited nucleotide pool. When the nucleic acid level was slightly increased but the toxic levels were not reached, animals showed a slight inhibition of reproductive signals and achieved lifespan extension. Certainly, the abnormal elevation of nucleotides could lead to the uncontrolled proliferation of cells, as previously mentioned [42]. These results indicated that as a nucleic acid receptor, the Notch signaling pathway could be downregulated under low nucleotide conditions, while under high nucleotide conditions, a negative feedback loop is activated to downregulate the Notch signaling pathway, ultimately maintaining nucleic acid homeostasis to maintain a normal physiological state (Supplemenary Figure 5).

UP is a member of the pyrimidine biosynthesis family of enzymes and is a key regulator of uridine homeostasis. The *C. elegans* UP homologs protein (UPP-1) exhibited both uridine and thymidine phosphorylase activity *in vitro*. UP activity is typically upregulated in various tumor tissues [46]. A previous study found that the lifespan of *upp-1* mutant worms was reduced by approximately 30% compared with that of wild-type worms [47]. However, in contrast, we observed a lifespan extension phenotype instead of a lifespan reduction phenotype following *upp-1* RNAi (Figure 2B). To confirm this difference, we obtained the *upp-1* (*jg1, jg2* and *jg3*) mutant strains from the Jaegal Shim laboratory and verified that the lifespan of the *upp-1* mutant worms was shortened (Supplementary Figure 3C). In addition, we constructed another *upp-1* RNAi vector (*upp-1^(##)^*) and detected lifespan when performing *upp-1^(##)^* RNAi. Similar to the result of *upp-1* RNAi clones (from the Ahringer libraries), animal exposed reconstructed *upp-1^(##)^* RNAi displayed the phenotype of lifespan extension (Supplementary Figure 3A). These two different phenotypes of *upp-1* RNAi and *upp-1* mutants may be caused by two mechanisms: first, the differences in the food source, HT115 (current study) versus OP50 [47] may contribute to these differences in phenotype [48, 49]; second and most importantly, in the previous study, authors used missense or nonsense *upp-1* mutants, which may cause the pyrimidine metabolite to reach an abnormally high level that leads to an abnormal physiological state. In our study, the RNAi treatment might cause a moderate decrease in *upp-1* function causing the beneficial accumulation of the intermediates but not at a toxic level that affects the physiological state. This accumulation might activate a negative feedback regulation loop to inhibit the Notch signaling pathway to achieve the increased longevity of *C. elegans* (Supplementary Figure 5).

Longevity molecules that prevent or attenuate age-related degeneration have long been the ultimate goal of humanity. Many studies have determined that nutrients including, carbohydrates, lipids, proteins, minerals, and vitamins, are essential for the survival of organisms. For example, a previous study reported that increasing glucose intake accelerates aging in yeast and *C. elegans* [50, 51]. Other researchers found that methionine restriction extends the lifespan of *Drosophila* by downregulating TOR signaling [52]. As one nutrient class, the effect of nucleotides on aging regulation has been scarcely reported. Here, we found that elevated concentrations of some nucleotide bases (e.g., thymine, orotate, β-aminoisobutyrate, uridine, and cytidine) extend the lifespan of worms. Endogenous metabolites such as pyrimidine intermediates could alter *C. elegans* lifespan, suggesting that an existing internal mechanism may be accessible to intervention; whether this mechanism can translate into manipulating the aging process in humans requires further investigation. The role of Notch signaling in modulating germline proliferation and longevity in response to changes in nucleotide abundance could prompt future mechanistic studies to discover the connections between nucleotide levels and Notch activity, which will help us not only better understand the detail of lifespan regulation by reproductive signals, but also propose a new avenue for investigating lifespan regulation mechanisms by interfering with endogenous metabolic pathways.

## MATERIALS AND METHODS

### Chemicals and strains

The strains used for this publication were obtained from the *Caenorhabditis* Genetics Center (CGC) (University of Minnesota, USA). Worms were maintained on nematode growth media (NGM) plates at 20 °C with *E. coli* OP50 bacteria as previously described unless otherwise stated [53]. All of the strains used in this study were: Bristol N2 wild-type, CF1038 *daf-16(mu86)I*, VC199 *sir-2.1(ok434)IV*, DA1116 *eat-2(ad1116)II*, CB1370 *daf-2(e1370)III*, AA89 *daf-12(rh274) X*, VC870 *nhr-49(gk405) I*, CF1903 *glp-1(e2144) III* and EU1 *skn-1(zu67) IV*.

All chemicals were purchased from Sigma-Aldrich (Munich, Germany), and resolved in PBS. NGM plates containing compounds were equilibrated overnight before use.

### RNA interference experiments

For RNAi gene knockdown experiments, we used *E. coli* HT115 as the food source for *C. elegans* as previously described [54]. The clones for *dpyd-1*, *upp-1*, *dhod-1*, and *upb-1* were derived from the Ahringer library (Source Bioscience, Nottingham, UK). For *upp-1^(##)^* RNAi, the designated fragment was obtained by amplification from genomic DNA with primers (forward, 5′- TTTTGACCCGTTAGTTGTCATGC -3′; reverse, 5′- ATCTCCATCCATACGATTCAAAAGA -3′) and subcloning into the L4440 feeding vector (pPD129.36). Then, the resulting plasmids were transformed into the HT115 (DE3) RNase III-deficient *E. coli* strain [55]. HT115 bacteria transformed with RNAi vectors (L4440) were grown at 37 °C in LB with 100 μg mL^-1^ ampicillin. Then, freshly prepared bacteria were spotted on NGM plates with 1 mM isopropyl-B-D-thiogalactoside (IPTG) and 100 μg mL^-1^ ampicillin. In experiments before treatment, synchronized L1 larvae were transferred to fresh plates with gene-specific RNAi bacteria.

### Lifespan assay

All strains were grown on NGM plates for 2-3 generations without starvation. All lifespan assays were performed at 20 °C except CF1903, according to standard protocols and as previously described [53]. In brief, 100 late L4 larvae or young adults were transferred to fresh plates containing 10 μM 5-fluro-2′-deoxyuridine (FUdR, Sigma) and the respective compounds and scored every day. To determine the lifespan of the temperature-sensitive mutant CF1903, L1 worms were incubated at 20 °C for 12 h, then transferred to 25 °C until L4 larvae or young adulthood, and then returned to 20 °C for the remainder of the lifespan [13]. To ensure drug potency, animals were transferred every second day. Experiments were repeated at least twice. The mean, SEM, *P* value and lifespan value were summarized in Supplementary Table 1 in the Supporting Information.

### Movement assay

Movement assays were performed as previously described [22, 53]. Briefly, 100 late L4 larvae or young adults were transferred to fresh plates with or without compound and maintained as described in the lifespan assay. When tapping plates, the worms moving in a continuous, coordinated sinusoidal way were characterized as fast movement; otherwise, the worms were classified as a nonfast movement.

### Fertility assay

Single L4 or young adults were transferred to single plates with or without compound and subsequently transferred every 24 h to a fresh plate. The offspring yielded by each worm on each day were allowed to hatch and were counted. For every experiment, more than 30 worms were used, and the experiments were conducted three times.

### Quantitative RT-PCR assay

Total RNA was extracted using RNAiso Plus (Takara) based on the phenol-chloroform extraction method. Afterward, the RNA was quantified and converted cDNAs using a high capacity cDNA transcription kit (RK20400, ABclonal) following the manufacturers' protocol. mRNA levels were quantified in a SYBR Green Select Master Mix (RK21203, ABclonal) on a CFX96 real-time system (Biorad). For every experiment, biological triplicates (starting from RNA isolation) and technical triplicates were performed. The results were calculated by the 2^-ΔΔ^Ct method, and normalized to the reference genes *cdc-42* [56]. *P* values were calculated using a two-tailed Student’s t test. The primers used in this publication are summarized in Supplementary Table 2 in the Supporting Information.

### Germ cell number quantification

Synchronized worms grown on plates with or without treatment were collected 12 h post-L4. Worms from each condition were washed three times with M9 buffer and dissected in M9 buffer with 5 mM imidazole under the dissection light microscope, transferred to a slide and freeze cracked. Gonads were stained using 4,6-diamidino-2-phenylindole (DAPI) as previously described with some modification [57]. Briefly, gonads were fixed in -20 °C MeOH for 10 min, washed twice in PBST (PBS with 0.1% Tween 20), and stained with 0.5 μg/ml DAPI for 10 min. Then, the gonads were washed twice in PBST before imaging. Imaging was performed with a Nikon Ti2-U microscope, and germ cell nuclei were manually counted from the distal tip to the beginning of meiotic entry. At least 20 animals were used for each experiment, and each experiment was repeated at least twice.

### Oil red O staining and quantification

ORO staining of fixed worms was conducted as previously described [58]. ORO-stained worms were mounted onto 2% agar pads and imaged at 20× magnification using a Nikon Ti2-U fluorescence microscope. The ORO intensity per worm was measured using Image-Pro-Plus processing software. Mean intensity values in arbitrary units (a.u.) were graphed using GraphPad Prism, and statistical significance was determined using a two-tailed Student’s t test. At least 30 animals were used for each experiment, and each experiment was repeated at least twice.

### Green fluorescent protein quantification and visualization

For the quantification of SOD-3 and GST-4, synchronized L1 larvae of CF1553 (muIs84 [Psod-3::GFP, rol-6]) and CL2166 (dvIs19 [(pAF15)gst-4p::GFP::NLS]) were transferred to compounds treated and untreated plates for 55 h. Worms were anesthetized in M9 containing 10 μM levamisole and mounted on 2% agar pads. The GFP fluorescence of worms was directly observed with a Nikon Ti2-U fluorescence microscope. At least 30 animals were used for each experiment. For GFP quantification, images were analyzed by ImageJ.

### Determination of pyrimidine intermediates concentrations by HPLC/MS

The strains were cultured for 2-3 generations prior to collection. The samples were prepared as previously described [13]. For sample treated with metabolites, synchronized L1 larvae were grown at the plates with or without metabolites until young adult for harvest. For *glp-1* mutants, synchronized L1 larvae were incubated at 20 °C for 12 h, then transferred to 25 °C to eliminate germ cells, until young adult stage for harvest, as well as corresponding control samples were treated via the same procedures. All samples were collected through M9 buffer, and then immediately flash frozen in liquid nitrogen and stored at -80°C until extraction.

Metabolites form *C. elegans* samples were extracted four times with 800 μL of precooled MeOH / H2O (2:1) using a TissueLyser at 75 Hz for 120 s. All extracts were subjected to centrifugation (12000 rpm for 10 min at 4 °C), supernatant was evaporated to dryness with a vacuo at a low temperature and dissolved in 400 μL MeOH / H2O (2:1) for LC/MS analysis.

Liquid chromatography was performed using a reversed-phase C18 column (Eclipse Plus C18, Agilent, 5μm, 150×2.1 mm diameter column) with a flow rate of 300 μL/min at room temperature, and 3μL of sample was injected. Eluent A was ACN, eluent B was H_2_O and eluent C was MeOH. The initial eluent consisted of 1% solvent A and 1% solvent C; the percent of buffer A and C were then gradually increased to 10% in 2 min, held there for 4 min, and then returned to the initial condition in 0.1 min, and held there for 10 min, the total run time was 16 min. Analyses were conducted using an Agilent Technologies 1260 (Agilent, Santa Clara, CA) system connected to an AB SCIEX QTRAP 4500 (ESI-MS/MS; Applied Biosystems, Foster City, CA).

Mass spectrometry analyses were conducted in negative ion multiple reaction monitoring (MRM) mode. The MS/MS parameters were optimized by infusion of individual compounds into the MS through a flow injection system.

## Supplementary Material

Supplementary Figures

Supplementary Table 1

Supplementary Table 2
