# Supplementary material for: Intermediate metabolites of the pyrimidine metabolism pathway extend the lifespan of C. elegans through regulating reproductive signals
Source: Aging (Albany NY). 2019 Jun 21;11(12):3993–4010. doi: 10.18632/aging.102033 (PMC6629003; doi:10.18632/aging.102033)
Supplement: Supplementary Figures [file aging-11-102033-s003.pdf]

SUPPLEMENTARY FIGURES

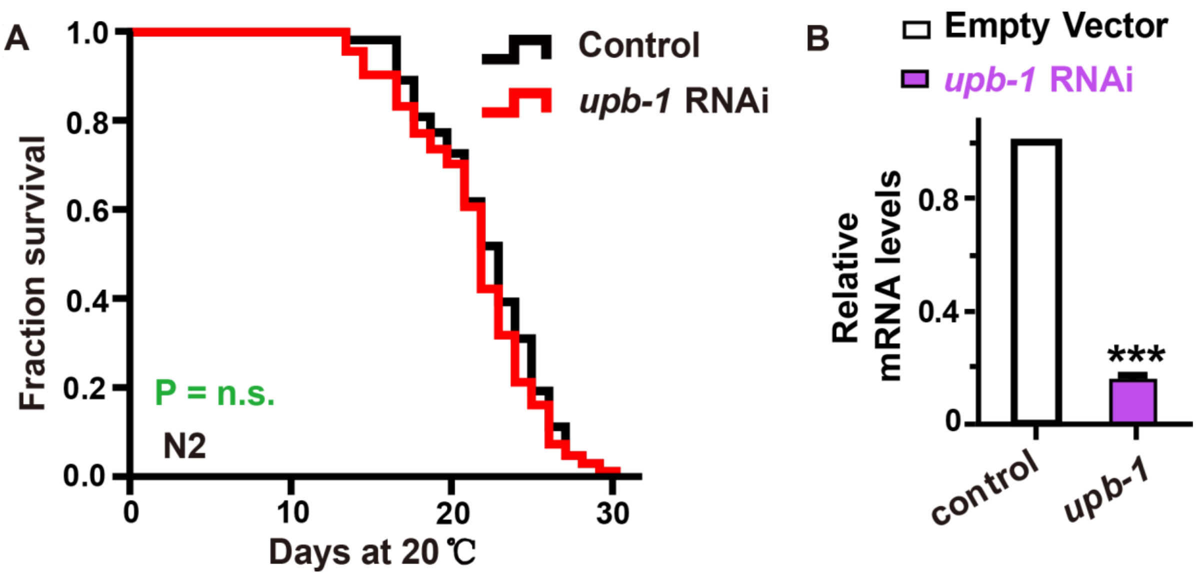

Supplementary Figure 1. Lifespan of wildtype animals exposed to *upb-1* RNAi bacteria. Lifespan analysis of wild-type N2 exposed to *upb-1* RNAi (red) or control bacteria (black). For *P* value, lifespan values of repetitions showed in supporting Supplementary Table 1. (**B**) *upb-1* RNA levels in whole worm RNA extracts after treatment of *C. elegans* with RNAi *upb-1* versus control RNAi. (mean ± SD of three independent experiments, each with three technical replicates, *** *P*<0.001, Student's t test).

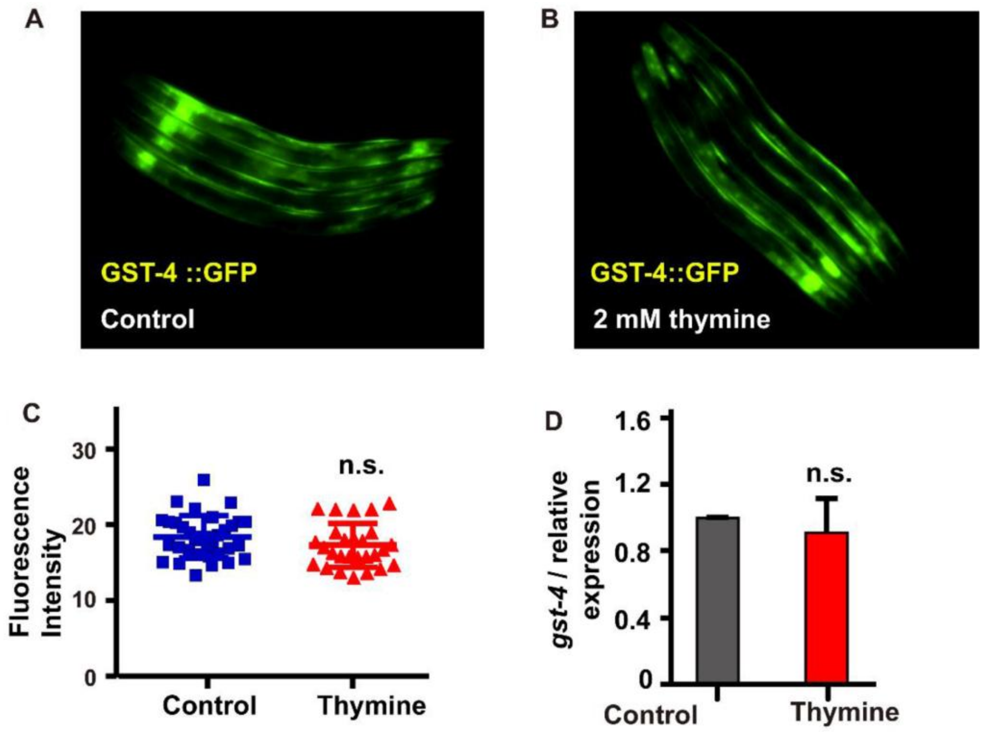

Supplementary Figure 2. Effect of thymine on the level of *gst-4*. (**A**–**C**) Images and quantification of GFP fluorescence. mean ± SD, n ≥ 30 (Student's t test). (**D**) QPCR analysis of the mRNA level of *gst-4*, means ± SD, n=3 (Student's t test).

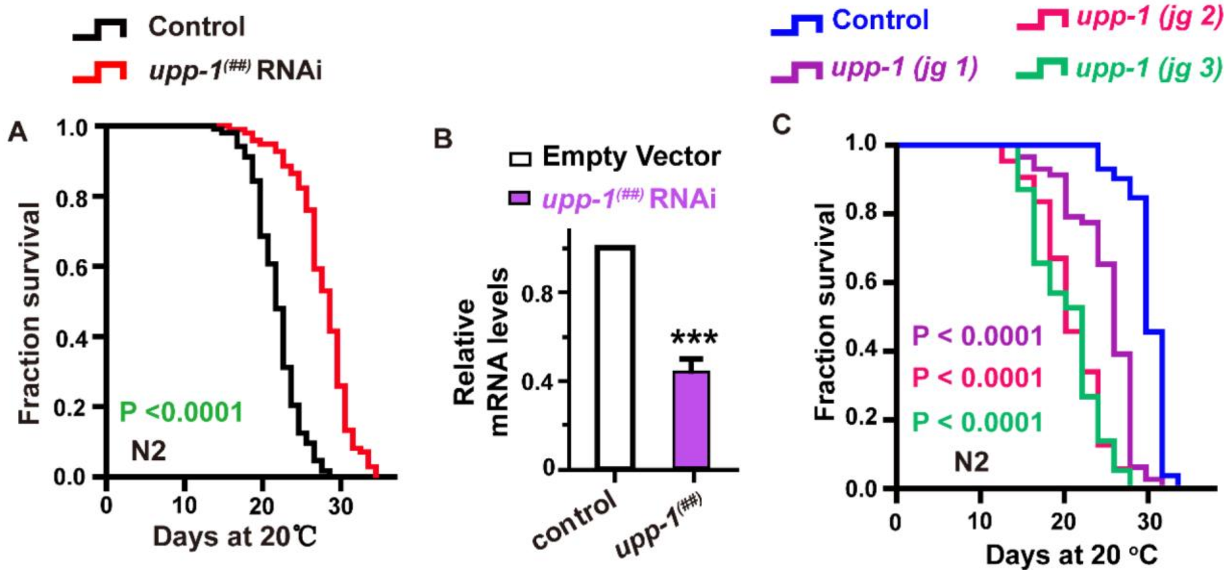

Supplementary Figure 3. Effect of *upp-1* on the aging of *Caenorhabditis elegans*. (**A**) Lifespan analysis of wild-type N2 exposed to *upp-1*$^{(\#\#)}$ RNAi (red) or control bacteria (black). For *P* value, lifespan values of repetitions showed in supporting Supplementary Table 1. (**B**) *upp-1* RNA levels after treatment of *C. elegans* with RNAi *upp-1*$^{(\#\#)}$ versus control RNAi. (mean ± SD of four independent experiments, each with three technical replicates, *** *P*<0.001, Student's t test). (**C**) Lifespan analysis of *upp-1 (jg1, jg2* and *jg3)* mutants. *P* value, lifespan values of repetitions showed in supporting Supplementary Table 1.

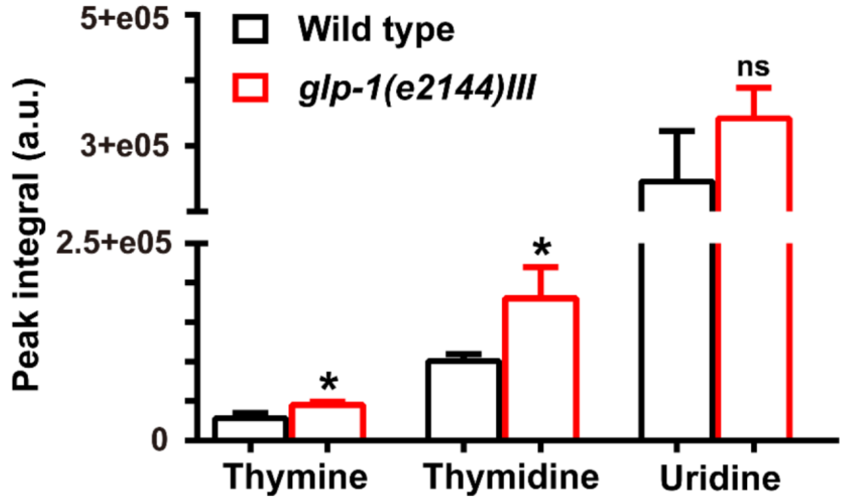

Supplementary Figure 4. Difference of concentration of pyrimidine intermediates between *glp-1* mutant and wild-type.

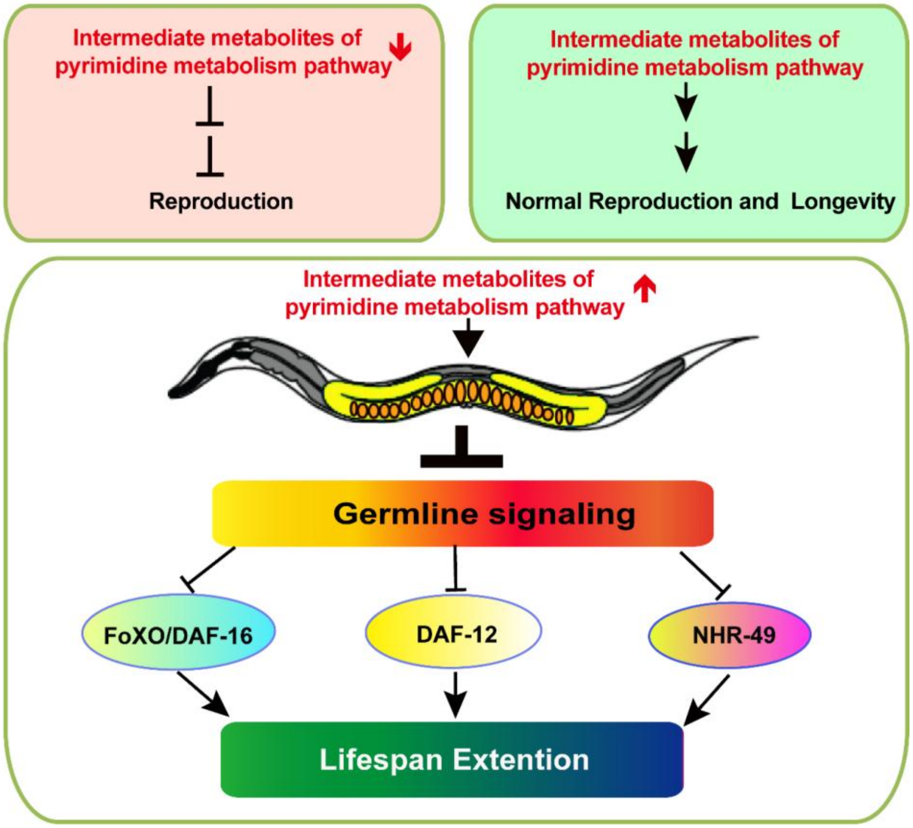

Supplementary Figure 5. Mechanisms of action of pyrimidine intermediates in *C. elegans.*
