# Supplementary material for: Intermediate metabolites of the pyrimidine metabolism pathway extend the lifespan of C. elegans through regulating reproductive signals
Source: Aging (Albany NY). 2019 Jun 21;11(12):3993–4010. doi: 10.18632/aging.102033 (PMC6629003; doi:10.18632/aging.102033)
Supplement: Supplementary Table 1 [file aging-11-102033-s001.docx]

**Supplementary Table 1. Survival analyses are conducted using the Kaplan-Meier method.**

| **Figure** | **Strains** | **Treatments** | **Mean**  **Lifespan ± SEM**  **(days)** | ***P* value**  **VS**  **control** | **%**  **Change in mean lifespan** | **N** |
| --- | --- | --- | --- | --- | --- | --- |
| **N2 (WT)** | | | | | | |
| **Figure 1D** | EXP. 1 | 20°C / control | 18.478±0.296 |  |  | 136 |
|  | EXP. 1 | 20°C/ 0.5 mM thymine | 21.951±0.390 | <0.0001 | 18.8 | 82 |
|  | EXP. 2 | 20°C/ control | 16.970±0.351 |  |  | 66 |
|  | EXP. 2 | 20°C/ 0.5 mM thymine | 19.367±0.513 | <0.0001 | 14.1 | 60 |
|  | EXP. 3 | 20°C/ control | 22.01±0.402 |  |  | 81 |
|  | EXP. 3 | 20°C/ 0.5 mM thymine | 25.03±0.685 | 0.001 | 12.8 | 82 |
|  |  |  |  |  |  |  |
| **Figure 1B** | EXP. 1 | 20°C/ control | 21.117±0.245 |  |  | 128 |
|  | EXP. 1 | 20°C/ 0.5 mM β-ami | 21.850±0.315 | 0.003 | 3.5 | 167 |
|  | EXP. 2 | 20°C/ control | 20.415±0.272 |  |  | 188 |
|  | EXP. 2 | 20°C/ 0.5 mM β-ami | 23.472±0.305 | <0.0001 | 15.0 | 180 |
|  | EXP. 3 | 20°C/ control | 23.846±0.404 |  |  | 74 |
|  | EXP. 3 | 20°C/ 0.5 mM β-ami | 26.379±0.308 | <0.0001 | 10.6 | 87 |
|  |  |  |  |  |  |  |
| **Figure 1C** | EXP. 1 | 20°C/ control | 21.117±0.245 |  |  | 128 |
|  | EXP. 1 | 20°C/ 0.5 mM orotate | 23.319±0.381 | <0.0001 | 10.4 | 135 |
|  | EXP. 2 | 20°C/ control | 20.286±0.318 |  |  | 105 |
|  | EXP. 2 | 20°C/ 0.5 mM orotate | 24.538±0.356 | <0.0001 | 21.0 | 106 |
|  | EXP. 3 | 20°C/ control | 20.973±0.477 |  |  | 73 |
|  | EXP. 3 | 20°C/ 0.5 mM orotate | 23.939±0.485 | 0.001 | 14.1 | 49 |
|  | EXP. 4 | 20°C/ control | 22.946±0.404 |  |  | 74 |
|  | EXP. 4 | 20°C/ 0.5 mM orotate | 26.092±0.409 | 0.0017 | 13.7 | 65 |
|  |  |  |  |  |  |  |
| **Figure 1F** | EXP. 1 | 20°C/ control ***** | 20.156±0.426 |  |  | 64 |
|  | EXP. 1 | 20°C/ 0.5 mM cytidine ***** | 22.366±0.379 | <0.0001 | 10.9 | 82 |
|  | EXP. 2 | 20°C/ control ***** | 19.908±0.345 |  |  | 87 |
|  | EXP. 2 | 20°C/ 0.5 mM cytidine ***** | 21.210±0.357 | 0.006 | 4.4 | 100 |
|  | EXP. 3 | 20°C/ control ***** | 19.361±0.472 |  |  | 61 |
|  | EXP. 3 | 20°C/ 0.5 mM cytidine ***** | 20.959±0.479 | 0.028 | 8.3 | 49 |
|  |  |  |  |  |  |  |
| **Figure 1E** | EXP. 1 | 20°C/ control ***** | 20.156±0.426 |  |  | 64 |
|  | EXP. 1 | 20°C/ 0.5 mM uridine ***** | 22.954±0.324 | <0.0001 | 13.9 | 65 |
|  | EXP. 2 | 20°C/ control ***** | 19.908±0.345 |  |  | 87 |
|  | EXP. 2 | 20°C/ 0.5 mM uridine ***** | 21.639±0.321 | <0.0001 | 8.7 | 147 |
|  | EXP. 3 | 20°C/ control ***** | 20.540±0.350 |  |  | 88 |
|  | EXP. 3 | 20°C/ 0.5 mM uridine ***** | 21.954±0.421 | 0.008 | 6.9 | 124 |
| **Figure 1H** | EXP. 1 | 20°C/ control | 20.973±0.477 |  |  | 73 |
|  | EXP. 1 | 20°C/ 0.5 mM uracil | 22.016±0.429 | 0.341 | # | 61 |
|  | EXP. 2 | 20°C/ control | 23.946±0.404 |  |  | 74 |
|  | EXP. 2 | 20°C/ 0.5 mM uracil | 23.474±0.404 | 0.468 | # | 76 |
|  | EXP. 3 | 20°C/ control | 24.603±0.387 |  |  | 58 |
|  | EXP. 3 | 20°C/ 0.5 mM uracil | 23.577±0.387 | 0.037 | # | 71 |
|  |  |  |  |  |  |  |
| **Figure 1G** | EXP. 1 | 20°C/ control | 21.117±0.245 |  |  | 128 |
|  | EXP. 1 | 20°C/ 0.5 mM thymidine | 21.619±0.292 | 0.126 | # | 105 |
|  | EXP. 2 | 20°C/ control | 20.054±0.214 |  |  | 204 |
|  | EXP. 2 | 20°C/ 0.5 mM thymidine | 20.592±0.219 | 0.333 | # | 142 |
|  | EXP. 3 | 20°C/ control | 20.286±0.318 |  |  | 105 |
|  | EXP. 3 | 20°C/ 0.5 mM thymidine | 20.867±0.255 | 0.294 | # | 135 |
|  |  |  |  |  |  |  |
| **Figure 3A** | EXP. 1 | 20°C/ control | 20.729±0.253 |  |  | 133 |
|  | EXP. 1 | 20°C/ 10 mM thymine | 22.213±0.428 | 0.028 | 7.2 | 150 |
|  | EXP. 1 | 20°C/ 2 mM thymine | 24.213±0.308 | <0.0001 | 16.8 | 150 |
|  | EXP. 1 | 20°C/ 0.5 mM thymine | 23.194±0.464 | <0.0001 | 11.9 | 72 |
|  | EXP. 1 | 20°C/ 0.1 mM thymine | 20.486±0.298 | 0.599 | # | 138 |
|  | EXP. 1 | 20°C/ 0.02 mM thymine | 19.617±0.272 | 0.01 | # | 133 |
|  | EXP. 2 | 20°C/ control | 23.133±0.451 |  |  | 98 |
|  | EXP. 2 | 20°C/ 10 mM thymine | 24.691±0.491 | 0.009 | 6.7 | 110 |
|  | EXP. 2 | 20°C/ 2 mM thymine | 27.116±0.426 | <0.0001 | 18.8 | 146 |
|  | EXP. 2 | 20°C/ 0.5 mM thymine | 24.696±0.399 | 0.021 | 6.7 | 125 |
|  | EXP. 2 | 20°C/ 0.1 mM thymine | 22.983±0.257 | 0.678 | # | 163 |
|  | EXP. 2 | 20°C/ 0.02 mM thymine | 23.256±0.478 | 0.781 | # | 139 |
|  | EXP. 3 | 20°C/ control | 22.213±0.827 |  |  | 61 |
|  | EXP. 3 | 20°C/ 10 mM thymine | 23.152±0.583 | 0.034 | 4.2 | 105 |
|  | EXP. 3 | 20°C/ 2 mM thymine | 26.528±0.798 | <0.0001 | 19.4 | 106 |
|  | EXP. 3 | 20°C/ 0.5 mM thymine | 25.233±0.661 | <0.0001 | 13.5 | 86 |
|  | EXP. 3 | 20°C/ 0.1 mM thymine | 22.456±0.711 | 0.776 | # | 89 |
|  | EXP. 3 | 20°C/ 0.02 mM thymine | 21.935±0.827 | 0.569 | # | 97 |
|  |  |  |  |  |  |  |
| **Figure 2C** | EXP. 1 | 20°C/ control | 19.259±0.247 |  |  | 147 |
|  | EXP. 1 | 20°C/ *dhod-1* RNAi | 16.678±0.174 | <0.0001 | -13.4 | 149 |
|  | EXP. 2 | 20°C/ control | 17.210±0.333 |  |  | 119 |
|  | EXP. 2 | 20°C/ *dhod-1* RNAi | 14.596±0.154 | <0.0001 | -15.2 | 146 |
|  | EXP. 3 | 20°C/ control | 23.264±0.313 |  |  | 163 |
|  | EXP. 3 | 20°C/ *dhod-1* RNAi | 21.878±0.306 | <0.0001 | -6.0 | 115 |
|  |  |  |  |  |  |  |
| **Figure 2C** | EXP. 1 | 20°C/ control | 21.544±0.628 |  |  | 68 |
|  | EXP. 1 | 20°C/ *dhod-1* RNAi | 19.628±0.360 | 0.005 | -8.9 | 94 |
|  | EXP. 1 | 20°C/ *dhod-1* RNAi +2 mM orotate | 21.949±0.378 | 0.771 | # | 79 |
|  | EXP. 2 | 20°C/ control | 21.633±0.345 |  |  | 94 |
|  | EXP. 2 | 20°C/ *dhod-1* RNAi | 20.181±0.302 | <0.0001 | -6.7 | 101 |
|  | EXP. 2 | 20°C/ *dhod-1* RNAi +2 mM orotate | 21.235±0.332 | 0.327 | # | 85 |
|  | EXP. 3 | 20°C/ control | 21.364±0.329 |  |  | 110 |
|  | EXP. 3 | 20°C/ *dhod-1* RNAi | 18.880±0.278 | <0.0001 | -11.6 | 92 |
|  | EXP. 3 | 20°C/ *dhod-1* RNAi +2 mM orotate | 20.766±0.308 | 0.089 | # | 94 |
|  |  |  |  |  |  |  |
| **Figure 2A** | EXP. 1 | 20°C/ control | 20.013±0.244 |  |  | 154 |
|  | EXP. 1 | 20°C/ *dpyd-1* RNAi | 22.556±0.292 | <0.0001 | 12.7 | 126 |
|  | EXP. 2 | 20°C/control | 18.898±0.299 |  |  | 98 |
|  | EXP. 2 | 20°C/ *dpyd-1* RNAi | 22.533±0.358 | <0.0001 | 19.2 | 122 |
|  | EXP. 3 | 20°C/ control | 23.264±0.313 |  |  | 163 |
|  | EXP. 3 | 20°C/ *dpyd-1* RNAi | 25.621±0.365 | <0.0001 | 10.1 | 116 |
|  | EXP. 4 | 20°C/ control | 22.699±0.334 |  |  | 123 |
|  | EXP. 4 | 20°C/ *dpyd-1* RNAi | 25.218±0.393 | <0.0001 | 11.1 | 101 |
|  |  |  |  |  |  |  |
| **Figure 2B** | EXP. 1 | 20°C/ control | 20.013±0.244 |  |  | 154 |
|  | EXP. 1 | 20°C/ *upp-1* RNAi | 25.325±0.332 | <0.0001 | 26.5 | 117 |
|  | EXP. 2 | 20°C/ control | 19.259±0.247 |  |  | 147 |
|  | EXP. 2 | 20°C/ *upp-1* RNAi | 23.495±0.469 | <0.0001 | 22.0 | 109 |
|  | EXP. 3 | 20°C/ control | 18.898±0.299 |  |  | 98 |
|  | EXP. 3 | 20°C/ *upp-1* RNAi | 23.333±0.355 | <0.0001 | 23.5 | 102 |
|  | EXP. 4 | 20°C/ control | 23.264±0.313 |  |  | 163 |
|  | EXP. 4 | 20°C/ *upp-1* RNAi | 25.581±0.312 | <0.0001 | 10.0 | 155 |
|  | EXP. 5 | 20°C/ control | 22.699±0.334 |  |  | 123 |
|  | EXP. 5 | 20°C/ *upp-1* RNAi | 26.400±0.517 | <0.0001 | 13.7 | 84 |
|  |  |  |  |  |  |  |
| **Figure S3A** | EXP. 1 | 20°C/ control | 22.235±0.296 |  |  | 102 |
|  | EXP. 1 | 20°C/ *upp-1*^(##)^ RNAi | 28.198±0.381 | <0.0001 | 26.8 | 96 |
|  | EXP. 2 | 20°C/ control | 24.511±0.425 |  |  | 88 |
|  | EXP. 2 | 20°C/ *upp-1*^(##)^ RNAi | 29.500±0.330 | <0.0001 | 20.4 | 128 |
|  | EXP. 3 | 20°C/ control | 23.817±0.370 |  |  | 104 |
|  | EXP. 3 | 20°C/ *upp-1*^(##)^ RNAi | 27.323±0.357 | <0.0001 | 14.7 | 124 |
|  | EXP. 4 | 20°C/ control | 20.269±0.363 |  |  | 104 |
|  | EXP. 4 | 20°C/ *upp-1*^(##)^ RNAi | 22.612±0.339 | <0.0001 | 11.6 | 116 |
|  |  |  |  |  |  |  |
|  |  |  |  |  |  |  |
| **Figure S3C** | EXP. 1 | 20°C/ control | 28.210±0.24 |  |  | 81 |
|  | EXP. 1 | 20°C/ *upp-1 (jg 1)* | 26.018±0.240 | <0.0001 | -8.4 | 114 |
|  | EXP. 1 | 20°C/ *upp-1 (jg 2)* | 21.851±0.509 | <0.0001 | -22.5 | 94 |
|  | EXP. 1 | 20°C/ *upp-1 (jg 3)* | 23.054±0.491 | <0.0001 | -18.3 | 74 |
|  |  |  |  |  |  |  |
|  | EXP. 2 | 20°C/ control | 31.310±0.274 |  |  | 71 |
|  | EXP. 2 | 20°C/ *upp-1 (jg 1)* | 26.797±0.483 | <0.0001 | -14.4 | 59 |
|  | EXP. 2 | 20°C/ *upp-1 (jg 2)* | 21.619±0.435 | <0.0001 | -30.9 | 84 |
|  | EXP. 2 | 20°C/ *upp-1 (jg 3)* | 21.087±0.625 | <0.0001 | -32.7 | 92 |
|  |  |  |  |  |  |  |
|  | EXP. 3 | 20°C/ control | 29.527±0.191 |  |  | 129 |
|  | EXP. 3 | 20°C/ *upp-1 (jg 1)* | 25.807±0.711 | <0.0001 | -12.6 | 57 |
|  | EXP. 3 | 20°C/ *upp-1 (jg 2)* | 22.469±0.345 | <0.0001 | -23.9 | 98 |
|  | EXP. 3 | 20°C/ *upp-1 (jg 3)* | 20.641±0.473 | <0.0001 | -30.1 | 78 |
|  |  |  |  |  |  |  |
|  | EXP. 4 | 20°C/ control | 28.663±0.304 |  |  | 89 |
|  | EXP. 4 | 20°C/ *upp-1 (jg 1)* | 25.947±0.539 | <0.0001 | -9.5 | 57 |
|  | EXP. 4 | 20°C/ *upp-1 (jg 2)* | 22.682±0.377 | <0.0001 | -20.9 | 88 |
|  | EXP. 4 | 20°C/ *upp-1 (jg 3)* | 21.343±0.556 | <0.0001 | -25.5 | 70 |
|  |  |  |  |  |  |  |
| **Figure S1A** | EXP. 1 | 20°C/ control | 21.544±0.628 |  |  | 68 |
|  | EXP. 1 | 20°C/ *upb-1* RNAi | 22.907±0.437 | 0.056 | # | 86 |
|  | EXP. 2 | 20°C/ control | 21.633±0.345 |  |  | 94 |
|  | EXP. 2 | 20°C/ *upb-1* RNAi | 21.354±0.356 | 0.510 | # | 82 |
|  | EXP. 3 | 20°C/ control | 21.364±0.329 |  |  | 110 |
|  | EXP. 3 | 20°C/ *upb-1* RNAi | 20.667±0.348 | 0.246 | # | 114 |
|  |  |  |  |  |  |  |
| ***daf-16(mu86)Ⅰ*** | | | | | | |
| **Figure 3C** | EXP. 1 | 20°C/ control | 20.850±0.165 |  |  | 120 |
|  | EXP. 1 | 20°C/ 2 mM thymine | 20.457±0.239 | 0.13 | # | 81 |
|  | EXP. 2 | 20°C/ control | 19.648±0.224 |  |  | 91 |
|  | EXP. 2 | 20°C/ 2 mM thymine | 19.430±0.212 | 0.41 | # | 100 |
|  | EXP. 3 | 20°C/ control | 19.535±0.185 |  |  | 127 |
|  | EXP. 3 | 20°C/ 2 mM thymine | 18.965±0.193 | 0.063 | # | 142 |
|  | EXP. 4 | 20°C/ control | 19.221±0.294 |  |  | 68 |
|  | EXP. 4 | 20°C/ 2 mM thymine | 19.556±0.286 | 0.475 | # | 63 |
|  |  |  |  |  |  |  |
| ***nhr-49(gk405) I*** | | | | | | |
| **Figure 3E** | EXP. 1 | 20°C/ control | 19.216±0.216 |  |  | 125 |
|  | EXP. 1 | 20°C/ 2 mM thymine | 19.907±0.218 | 0.301 | # | 108 |
|  | EXP. 2 | 20°C/ control | 19.529±0.218 |  |  | 119 |
|  | EXP. 2 | 20°C/ 2 mM thymine | 19.207±0.215 | 0.236 | # | 111 |
|  | EXP. 3 | 20°C/ control | 19.724±0.192 |  |  | 98 |
|  | EXP. 3 | 20°C/ 2 mM thymine | 19.970±0.172 | 0.591 | # | 99 |
|  |  |  |  |  |  |  |
| ***skn-1(zu67) IV*** | | | | | | |
| **Figure 3F** | EXP. 1 | 20°C/ control | 20.444±0.433 |  |  | 90 |
|  | EXP. 1 | 20°C/ 2 mM thymine | 21.873±0.482 | <0.0001 | 6.9 | 79 |
|  | EXP. 2 | 20°C/ control | 20.105±0.392 |  |  | 76 |
|  | EXP. 2 | 20°C/ 2 mM thymine | 21.848±0.416 | 0.005 | 8.6 | 92 |
|  | EXP. 3 | 20°C/ control | 20.694±0.367 |  |  | 96 |
|  | EXP. 3 | 20°C/ 2 mM thymine | 22.934±0.412 | <0.0001 | 10.8 | 103 |
|  |  |  |  |  |  |  |
| ***sir-2.1(ok434)Ⅳ*** | | | | | | |
| **Figure 4C** | EXP. 1 | 20°C/ control | 21.150±0.201 |  |  | 254 |
|  | EXP. 1 | 20°C/ 2 mM thymine | 22.310±0.222 | <0.0001 | 5.5 | 258 |
|  | EXP. 2 | 20°C/ control | 22.130±0.424 |  |  | 115 |
|  | EXP. 2 | 20°C/ 2 mM thymine | 23.840±0.475 | 0.014 | 7.73 | 109 |
|  | EXP. 3 | 20°C/ control | 19.053±0.203 |  |  | 225 |
|  | EXP. 3 | 20°C/ 2 mM thymine | 21.669±0.284 | <0.0001 | 13.7 | 175 |
|  |  |  |  |  |  |  |
| ***eat-2(ad1116)Ⅱ*** | | | | | | |
| **Figure 4B** | EXP. 1 | 20°C/ control | 25.512±0.348 |  |  | 86 |
|  | EXP. 1 | 20°C/ 2 mM thymine | 27.00±0.297 | 0.001 | 5.8 | 162 |
|  | EXP. 2 | 20°C/ control | 24.182±0.255 |  |  | 143 |
|  | EXP. 2 | 20°C/ 2 mM thymine | 27.378±0.249 | <0.0001 | 13.2 | 180 |
|  | EXP. 3 | 20°C/ control | 25.256±0.297 |  |  | 123 |
|  | EXP. 3 | 20°C/ 2 mM thymine | 27.476±0.261 | <0.0001 | 8.8 | 158 |
|  |  |  |  |  |  |  |
|  |  |  |  |  |  |  |
| ***daf-2(e1370)Ⅲ*** | | | | | | |
| **Figure 4A** | EXP. 1 | 20°C/ control | 52.402±0.661 |  |  | 127 |
|  | EXP. 1 | 20°C/ 2 mM thymine | 56.613±0.597 | <0.0001 | 8.0 | 119 |
|  | EXP. 2 | 20°C/ control | 52.379±0.537 |  |  | 116 |
|  | EXP. 2 | 20°C/ 2 mM thymine | 56.944±0.669 | <0.0001 | 8.7 | 125 |
|  | EXP. 3 | 20°C/ control | 52.544±0.479 |  |  | 169 |
|  | EXP. 3 | 20°C/ 2 mM thymine | 58.033±0.476 | <0.0001 | 10.5 | 209 |
|  | | | | | | |
| ***daf-12(rh274) X*** | | | | | | |
| **Figure 3C** | EXP. 1 | 20°C/ control | 25.099±0.555 |  |  | 86 |
|  | EXP. 1 | 20°C/ 2 mM thymine | 23.5±0.586 | 0.102 | # | 101 |
|  | EXP. 2 | 20°C/ control | 22.943±0.498 |  |  | 106 |
|  | EXP. 2 | 20°C/ 2 mM thymine | 23.957±0.567 | 0.139 | # | 93 |
|  | EXP. 3 | 20°C/ control | 22.211±0.472 |  |  | 90 |
|  | EXP. 3 | 20°C/ 2 mM thymine | 23.198±0.499 | 0.145 | # | 81 |
|  |  |  |  |  |  |  |
| **Figure 6A** | EXP. 1 | 20°C/ control | 21.603±0.585 |  |  | 58 |
|  | EXP. 1 | 20°C/ 0.5 mM β-ami | 21.424±0.585 | 0.905 | # | 66 |
|  | EXP. 2 | 20°C/ control | 23.333±0.589 |  |  | 87 |
|  | EXP. 2 | 20°C/ 0.5 mM β-ami | 22.982±0.671 | 0.602 | # | 55 |
|  | EXP. 3 | 20°C/ control | 23.841±0.563 |  |  | 63 |
|  | EXP. 3 | 20°C/ 0.5 mM β-ami | 23.763±0.531 | 0.677 | # | 76 |
|  |  |  |  |  |  |  |
| **Figure 6C** | EXP. 1 | 20°C/ control | 21.603±0.585 |  |  | 58 |
|  | EXP. 1 | 20°C/ 0.5 mM orotate | 22.785±0.590 | 0.091 | # | 65 |
|  | EXP. 2 | 20°C/ control | 23.333±0.589 |  |  | 87 |
|  | EXP. 2 | 20°C/ 0.5 mM orotate | 23.576±0.525 | 0.695 | # | 55 |
|  | EXP. 3 | 20°C/ control | 23.855±0.540 |  |  | 83 |
|  | EXP. 3 | 20°C/ 0.5 mM orotate | 23.3372±0.527 | 0.485 | # | 95 |
|  |  |  |  |  |  |  |
| **Figure 6B** | EXP. 1 | 20°C/ control ***** | 19.687±0.39 |  |  | 83 |
|  | EXP. 1 | 20°C/ 0.5 mM cytidine ***** | 19.551±0.396 | 0.748 | # | 78 |
|  | EXP. 2 | 20°C/ control ***** | 20.878±0.329 |  |  | 90 |
|  | EXP. 2 | 20°C/ 0.5 mM cytidine ***** | 20.909±0.392 | 0.99 | # | 66 |
|  | EXP. 3 | 20°C/ control ***** | 20.227±0.369 |  |  | 75 |
|  | EXP. 3 | 20°C/ 0.5 mM cytidine ***** | 19.983±0.411 | 0.703 | # | 59 |
|  | EXP. 4 | 20°C/ control ***** | 19.361±0.472 |  |  | 61 |
|  | EXP. 4 | 20°C/ 0.5 mM cytidine ***** | 20.424±0.450 | 0.132 | # | 59 |
|  |  |  |  |  |  |  |
|  |  |  |  |  |  |  |
| **Figure 6D** | EXP. 1 | 20°C/ control ***** | 19.687±0.39 |  |  | 83 |
|  | EXP. 1 | 20°C/ 0.5 mM uridine ***** | 19.573±0.353 | 0.644 | # | 89 |
|  | EXP. 2 | 20°C/ control ***** | 20.878±0.329 |  |  | 90 |
|  | EXP. 2 | 20°C/ 0.5 mM uridine ***** | 20.833±0.415 | 0.969 | # | 60 |
|  | EXP. 3 | 20°C/ control ***** | 20.227±0.369 |  |  | 75 |
|  | EXP. 3 | 20°C/ 0.5 mM uridine ***** | 19.310±0.394 | 0.101 | # | 58 |
|  |  |  |  |  |  |  |
| ***glp-1(e2144) III*** | | | | | | |
| **Figure 4D** | EXP. 1 | 20°C/ control | 31.719±0.499 |  |  | 128 |
|  | EXP. 1 | 20°C/ 2 mM thymine | 32.358±0.547 | 0.145 | # | 137 |
|  | EXP. 2 | 20°C/ control | 34.339±0.555 |  |  | 112 |
|  | EXP. 2 | 20°C/ 2 mM thymine | 32.436±0.593 | 0.077 | # | 117 |
|  | EXP. 3 | 20°C/ control | 34.601±0.451 |  |  | 168 |
|  | EXP. 3 | 20°C/ 2 mM thymine | 32.970±0.675 | 0.051 | # | 100 |
|  |  |  |  |  |  |  |
| **Figure 5A** | EXP. 1 | 20°C/ control | 33.172±0.796 |  |  | 93 |
|  | EXP. 1 | 20°C/ 0.5 mM β-ami | 33.232±0.761 | 0.891 | # | 99 |
|  | EXP. 2 | 20°C/ control | 36.118±0.649 |  |  | 93 |
|  | EXP. 2 | 20°C/ 0.5 mM β-ami | 36.782±0.630 | 0.447 | # | 101 |
|  | EXP. 3 | 20°C/ control | 31.064±0.838 |  |  | 78 |
|  | EXP. 3 | 20°C/ 0.5 mM β-ami | 29.905±0.520 | 0.07 | # | 95 |
|  |  |  |  |  |  |  |
| **Figure 5C** | EXP. 1 | 20°C/ control | 33.407±0.519 |  |  | 113 |
|  | EXP. 1 | 20°C/ 0.5 mM orotate | 34.230±0.515 | 0.14 | # | 122 |
|  | EXP. 2 | 20°C/ control | 33.172±0.796 |  |  | 93 |
|  | EXP. 2 | 20°C/ 0.5 mM orotate | 32.683±0.694 | 0.513 | # | 101 |
|  | EXP. 3 | 20°C/ control | 35.341±0.623 |  |  | 132 |
|  | EXP. 3 | 20°C/ 0.5 mM orotate | 35.539±0.469 | 0.086 | # | 128 |
|  | EXP. 4 | 20°C/ control | 34.182±0.739 |  |  | 110 |
|  | EXP. 4 | 20°C/ 0.5 mM orotate | 34.951±0.668 | 0.773 | # | 102 |
|  |  |  |  |  |  |  |
| **Figure 5B** | EXP. 1 | 20°C/ control ***** | 27.289±0.481 |  |  | 90 |
|  | EXP. 1 | 20°C/ 0.5 mM cytidine ***** | 26.916±0.468 | 0.714 | # | 83 |
|  | EXP. 2 | 20°C/ control ***** | 29.183±0.487 |  |  | 109 |
|  | EXP. 2 | 20°C/ 0.5 mM cytidine ***** | 28.211±0.544 | 0.420 | # | 109 |
|  | EXP. 3 | 20°C/ control ***** | 27.121±0.470 |  |  | 107 |
|  | EXP. 3 | 20°C/ 0.5 mM cytidine ***** | 27.991±0.450 | 0.196 | # | 106 |
|  | EXP. 4 | 20°C/ control ***** | 26.935±0.501 |  |  | 107 |
|  | EXP. 4 | 20°C/ 0.5 mM cytidine ***** | 26.519±0.574 | 0.984 | # | 104 |
|  |  |  |  |  |  |  |
| **Figure 5D** | EXP. 1 | 20°C/ control ***** | 29.183±0.487 |  |  | 109 |
|  | EXP. 1 | 20°C/ 0.5 mM uridine ***** | 27.723±0.523 | 0.056 | # | 94 |
|  | EXP. 2 | 20°C/ control ***** | 27.121±0.470 |  |  | 107 |
|  | EXP. 2 | 20°C/ 0.5 mM uridine ***** | 28.075±0.49 | 0.057 | # | 106 |
|  | EXP. 3 | 20°C/ control ***** | 26.935±0.501 |  |  | 107 |
|  | EXP. 3 | 20°C/ 0.5 mM uridine ***** | 27.827±0.601 | 0.205 | # | 81 |

*P* values were calculated for individual experiments by log-rank test. “N” in the table displayed the number of dead worms. All statistical were performed using SPSS package. “#”: no calculate (because p>0.05), β-ami: β-aminoisobutyrate, Asteriks (*) denotes lifespan analysis were conducted using NGM plates without FUdR.
