# Supplementary material for: Intermediate metabolites of the pyrimidine metabolism pathway extend the lifespan of C. elegans through regulating reproductive signals
Source: Aging (Albany NY). 2019 Jun 21;11(12):3993–4010. doi: 10.18632/aging.102033 (PMC6629003; doi:10.18632/aging.102033)
Supplement: Supplementary Table 2 [file aging-11-102033-s002.pdf]

Supplementary Table 2. Sequences of PCR primers (qPCR).

Primer sequences used for quantitative PCR (5′→3′):		
Gene	Forward primer sequence	Reverse primer sequence
cdc-42	CTGCTGGACAGGAAGATTACG	CTCGGACATTCTCGAATGAAG
sod-3	AGCATCATGCCACCTACGTGA	AGCATCATGCCACCTACGTGA
upb-1	TGGCGTTGAAACTGCTTTGG	AACGCGAACCAGTCTTGGAG
upp-1	GTGCGCCGACGATTTCTATG	TGCTTGCGAATGTAGGCAGT
dhod-1	AGCATCATGCCACCTACGTGA	GAATTTGTCCGTTTGTGAGTTTGT
dpyd-1	CAGTCACCCATCAGCCACAT	TTTGGAGTTCCTGGCTCGAC
cdr-6	TCGGGCTTCTCGGTTTACC	CAGCTTTGACCAGAGGAACCA
lips-17	ATCTGTTGCTGGAGCCAATCG	TATCCAACTTTATCGTCTCC
fard-1	GGGTTTTTGGGAAAGGTGAT	CCACCGATTGCTTTCAATTT
dod-3	CGTATATGGACCCAGCTAATG	ATGAACACCGGCTCATTC
lipl-4	AAAATCATGGGGTTACTCAGTTG	ATCCCAGCTCCAATCCCAAAA
acs-2	TGATGCTCATGTCGTCGGTG	TGACAGTTCCGAGACCCAAC
ech-1	GGGCAAAAGGCAGCAAGAAA	AATGGTGGGAAACCGAGACC
lbp-8	GATGGTGACACTTGGCATTTCA	TGTTGTAGGATCGCTCATCCG
